# Sporadic Creutzfeldt–Jakob disease infected human cerebral organoids retain the original human brain subtype features following transmission to humanized transgenic mice

**DOI:** 10.1186/s40478-023-01512-1

**Published:** 2023-02-14

**Authors:** Bradley R. Groveman, Brent Race, Simote T. Foliaki, Katie Williams, Andrew G. Hughson, Chase Baune, Gianluigi Zanusso, Cathryn L. Haigh

**Affiliations:** 1 Laboratory of Persistent Viral Diseases, Division of Intramural Research, Rocky Mountain Laboratories, National Institute of Allergy and Infectious Diseases, National Institutes of Health, 903 South 4th Street, Hamilton, MT 59840 USA; 2 Department of Neurosciences, Biomedicine and Movement Sciences, University of Verona, Verona, Italy

**Keywords:** Sporadic CJD, Subtype, Prion, PrP, Cerebral organoid

## Abstract

Human cerebral organoids (COs) are three-dimensional self-organizing cultures of cerebral brain tissue differentiated from induced pluripotent stem cells. We have recently shown that COs are susceptible to infection with different subtypes of Creutzfeldt–Jakob disease (CJD) prions, which in humans cause different manifestations of the disease. The ability to study live human brain tissue infected with different CJD subtypes opens a wide array of possibilities from differentiating mechanisms of cell death and identifying neuronal selective vulnerabilities to testing therapeutics. However, the question remained as to whether the prions generated in the CO model truly represent those in the infecting inoculum. Mouse models expressing human prion protein are commonly used to characterize human prion disease as they reproduce many of the molecular and clinical phenotypes associated with CJD subtypes. We therefore inoculated these mice with COs that had been infected with two CJD subtypes (MV1 and MV2) to see if the original subtype characteristics (referred to as strains once transmitted into a model organism) of the infecting prions were maintained in the COs when compared with the original human brain inocula. We found that disease characteristics caused by the molecular subtype of the disease associated prion protein were similar in mice inoculated with either CO derived material or human brain material, demonstrating that the disease associated prions generated in COs shared strain characteristics with those in humans. As the first and only in vitro model of human neurodegenerative disease that can faithfully reproduce different subtypes of prion disease, these findings support the use of the CO model for investigating human prion diseases and their subtypes.

**Supplementary Information:**

The online version contains supplementary material available at 10.1186/s40478-023-01512-1.

## Introduction

Prion diseases (PrD) are a family of fatal, neurodegenerative conditions that affect humans and animals. PrDs have three etiologies: hereditary, acquired by infection and sporadic (no known cause), of which the most common form is sporadic Creutzfeldt Jakob Disease (sCJD). Prions are misfolded conformers of the normal cellular prion protein (PrP^C^). Once formed, disease associated prions (PrP^D^) can recruit PrP^C^ causing it to also misfold and in this way propagate themselves in an ongoing cycle. sCJD, has several subtypes, as determined by biochemical signatures of the prion protein (PrP^D^) and the lesions formed within the brain [1–3]. The hallmark features of prion diseases include deposits of insoluble, protease resistant prion protein (PrP) isoforms (PrP^Res^), astrogliosis, and spongiform changes in the brain. However, the different subtypes manifest different symptoms in patients and follow different disease courses. The different symptomology and disease course of sCJD subtypes in humans has been attributed to selective neuronal vulnerability, where certain neuronal subsets are more vulnerable to attack by one sCJD subtype than by others [4]. Understanding neuronal selective vulnerability has been limited by the lack of human neuronal cultures that can propagate different human prion subtypes.

The basis for the different manifestations of the disease, caused by the same protein, is currently unknown but is thought to be encoded in slight structural differences within the PrP^D^ [5–8]. These subtype dependent structural changes are demonstrated by small shifts in the mobility of PrP^Res^ during electrophoresis and western blotting, for example type 1 or type 2 sCJD [3]. The PrP^Res^ banding pattern can be further characterized by the relative abundance of its three glycoforms, di-, mono- and un-glycosylated, with the mono-glycosylated band being dominant in sCJD brain. Furthermore, a polymorphism at codon 129 of the prion protein gene, encoding for either methionine (M) or valine (V), also influences disease course and susceptibility. Together, the M/V genotype and PrP^Res^ mobility pattern tend to couple with disease course and symptomology and are used to classify the different disease subtypes [1, 3, 9].

We previously sought to develop an in vitro human model of prion disease that could reproduce the subtype phenomena seen in the brain. To this end we generated cerebral organoids (COs) derived from human induced pluripotent stem cells ([10] and Additional File 1: Figure S1). COs are three-dimensional spheres of human brain tissue containing diverse cerebral cortex cell types and distinct neuronal populations [11, 12]. The COs were able to take up and clear the initial sCJD brain homogenate (BH) inocula then propagate de novo prions templated by the inoculating PrP^D^ (Additional File 1: Figure S1). Brain homogenates from two different sCJD subtypes were used and were classified as, MV1 and MV2 (using the classification system in [3, 9]). The organoids infected with the MV1 subtype showed only a small accumulation of prions, below the detection threshold of western blot, but demonstrated distinct changes in organoid health, more so than with MV2. The organoids infected with the subtype classified as MV2 accumulated prions that were detectible by western blotting and deposits were visualized by immunohistochemistry for prion protein within the organoids. However, the MV2 PrP^Res^ showed a changed electrophoretic profile from that expected: these organoids generated predominantly di-glycosylated PrP^Res^ rather than the expected mono-glycosylated dominant banding (which is characteristic of MV2 sCJD) ([10, 13] and Additional File 1: Figure S1). Di-glycosylated PrP^C^ is the primary glycoform in these organoids, so it is therefore not surprising that the PrP^Res^ deposited in them would be as well. However, since organoids offer many possible advantages over traditional cultures systems for investigating human prion disease [13–15], it was important to ascertain how accurately the organoids reproduced the original disease or whether the shift in glycosylation resulted from a tissue culture induced change in the propagating PrP^D^ analogous to a strain adaptation.

Strain adaptations often occur when prions are passaged through new hosts [16] where cellular differences such as cofactor availability [17] result in phenotypic change such as altered incubation time and PrP^D^ deposition [18]. For example, sheep scrapie passed through either mice or goats, and then mice, has given rise to multiple strains of rodent adapted scrapie, each with distinct clinical and biochemical manifestations [19]. The gold standard for investigating prion strain phenomenon remains mouse bioassay [20]. Transgenic mice can be made that are susceptible to a wide variety of prion strains and demonstrate remarkable reproducibility of disease phenotype for a given strain [21]. While passaging human prions into human tissue (cerebral organoids) does not necessarily constitute a host difference, the environment within the organoid may differ from an intact human brain. Therefore, to determine whether the prions produced by sCJD infection of organoids retain the features of the original inoculum or result in a strain adaptation, we performed bioassays using tg66 mice overexpressing human PrP^C^ [22]. Our findings demonstrated that the clinical and biochemical properties of the prions propagated in mice following inoculation with sCJD infected organoid homogenates were nearly indistinguishable from those inoculated with the original brain derived inocula. Additionally, the observed maintenance of the original sCJD subtypes after passage through the organoids indicates that organoids propagate prion disease with the attributes of the parent subtype without any apparent strain adaptation.

## Methods

### Experimental mice

Tg66 transgenic mice overexpressing human PrP^C^ were used for this study. These mice have been shown to be susceptible to prion disease by infection with MV1 and 2 sCJD prions, with distinct disease courses and biochemistry apparent with the different inocula [23]. Generation of these mice was described previously [22]. Tg66 mice were originally made by Richard Rubenstein and provided to RML by Robert Rohwer. Tg66 mice are on an FVB/N genetic background and are homozygous for a transgene that encodes human prion protein M129. Tg66 mice overexpress human PrP^C^ at 8–16-fold levels higher than normal physiologic levels and have been shown to be susceptible to vCJD, sCJD and mouse-adapted 22L scrapie [22, 24]. Tg66 mice do not express any normal mouse PrP^C^.

### Mouse inoculations and observations

Homogenates for inoculation were diluted from existing 10–20% whole organoid, human brain or mouse brain homogenates (Additional File 1: Figure S1). Briefly, aliquots of 10–20% homogenates in sterile phosphate buffered saline (PBS, pH 7.4) were sonicated for 1 min at a 60% amplitude setting. Following sonication, homogenates were further diluted to a 1% w/v concentration in phosphate buffered balanced saline supplemented with 2% fetal bovine serum. Mice were anesthetized with isoflurane and inoculated in the left brain hemisphere with 30 µl of 1% tissue homogenate. For the first passage experiments, 8–10 mice were inoculated for each experimental (homogenate) group, including MV1 or MV2 brain homogenate (MV-BH) or MV1 or MV2 infected CO homogenate (MV-OH). The second passage experiments included 8–12 mice per group inoculated with brain homogenate from mice inoculated with either BH or OH (MV-BH (ms) or MV-OH (ms)).

Post-inoculation, all mice were observed once daily by animal care staff and 3–5 times weekly by investigators for assessment of overall health and observation for signs consistent with prion infection. Mice inoculated with first passage MV1-BH, second passage MV1-BH (ms) or MV1-OH (ms) developed clinical signs including nesting deficiencies, gait abnormalities, weight loss, despondence, respiratory difficulty and tail wounding. The typical MV1 disease course was progressive and lasted 3–4 weeks. Mice were considered “clinical” and euthanized when they were nearing a moribund condition. Mice that were euthanized early or found dead due to non-TSE related reasons were excluded from the total clinical numbers. However, some tissues from excluded animals were still used for other analyses. Mice inoculated with first passage MV2-BH or OH and second passage MV2-BH or OH (ms) had a longer incubation period and the duration of clinical signs was much longer than MV1-infected mice, ranging 6–13 weeks. Common clinical signs in MV2 infected mice included weight loss, urinary bladder distention and incontinence, kyphosis, gait abnormalities, poor coat quality, crusted blood in the nares, progressive lethargy and somnolence.

### Real-time quaking-induced conversion (RT-QuIC) assay

RT-QuIC assays to measure prion seeding activity were performed as previously described [25, 26]. Briefly, 10% (w/v) pre-cleared BHs were serially diluted by 10 folds in 0.1% sodium dodecyl sulfate (SDS)/PBS/N2. Samples were tested in quadruplicate wells of a 384 well plate (Nunc). One µL of sample dilution was added to each well along with 49 µL of reaction mix (final concentrations of: 10 mM phosphate buffer [pH 7.4], 300 mM NaCl, 0.1 mg/mL hamster recombinant PrP 90–231, 10 μM thioflavin T (ThT), 1 mM ethylenediaminetetraacetic acid tetrasodium salt and 0.002% (w/v) SDS). Plates were sealed and incubated in a FLUOstar Omega plate reader (BMG) at 50 °C for 30 h with cycles of 60 s of shaking (700 rpm, double orbital) and 60 s of rest. ThT fluorescence was measured every 45 min (excitation, 450 ± 10 nm; emission, 480 ± 10 nm [bottom read]). As described previously, individual replicate wells were considered positive for seeding activity when the fluorescence value exceeded a threshold of 10% of the maximum value from any individual reaction well in the experiment within the 50 h time cutoff [10, 27, 28]. A sample was considered positive if greater than 25% of the reaction wells showed positive seeding activity. Data was plotted using GraphPad Prism 9.

### Western blotting

Brain homogenates in PBS containing 1% (v/v) sarkosyl were digested with 50 ug/ml PK at 37 °C for 1 h before boiling for 5 min in 1 × sample buffer (Invitrogen) containing 6% (v/v) 2-Mecaptoethanol. Samples were resolved in Bolt 4–12% Bis–Tris gels (Invitrogen) and transferred to PVDF membrane (Millipore). Membranes were blocked with EveryBlot Blocking Buffer (Biorad) for 10 min and incubated in anti-PrP 3F4 antibody (Millipore) at 1:10,000 dilution overnight. The protein bands were visualized using ECL Select (Amersham) and imaged using the iBright imaging system (Invitrogen).

### Immunohistochemistry, histology and lesion profiling

Brain fixation, paraffin embedding, H&E staining and immunohistochemistry for prion protein (biotinylated-3F4) were carried out as described previously [29]. Histopathology slides were analyzed by an observer blinded to the animal inoculation groups. Lesion profiling for spongiform pathology was completed on all available mice, as indicated in Tables 2 and 4. Nine brain regions (frontal cortex, hippocampus, striatum, thalamus, hypothalamus, colliculi, midbrain, pons/medulla, and cerebellum) were scored for spongiosis as follows: 0, no vacuoles; 1, few vacuoles widely and unevenly distributed; 2, few vacuoles evenly distributed; 3, moderate numbers of vacuoles evenly distributed; and 4, many vacuoles with some confluences. Lesion scores were graphed and presented using GraphPad prism.

### Statistical analysis

Statistical analysis, as indicated in figure legends, was carried out in GraphPad Prism. Lesion scores were compared by Mann–Whitney U test (*p* < 0.05).

## Results

### sCJD prions from infected organoids transmit to mice.

**Fig. 1:**
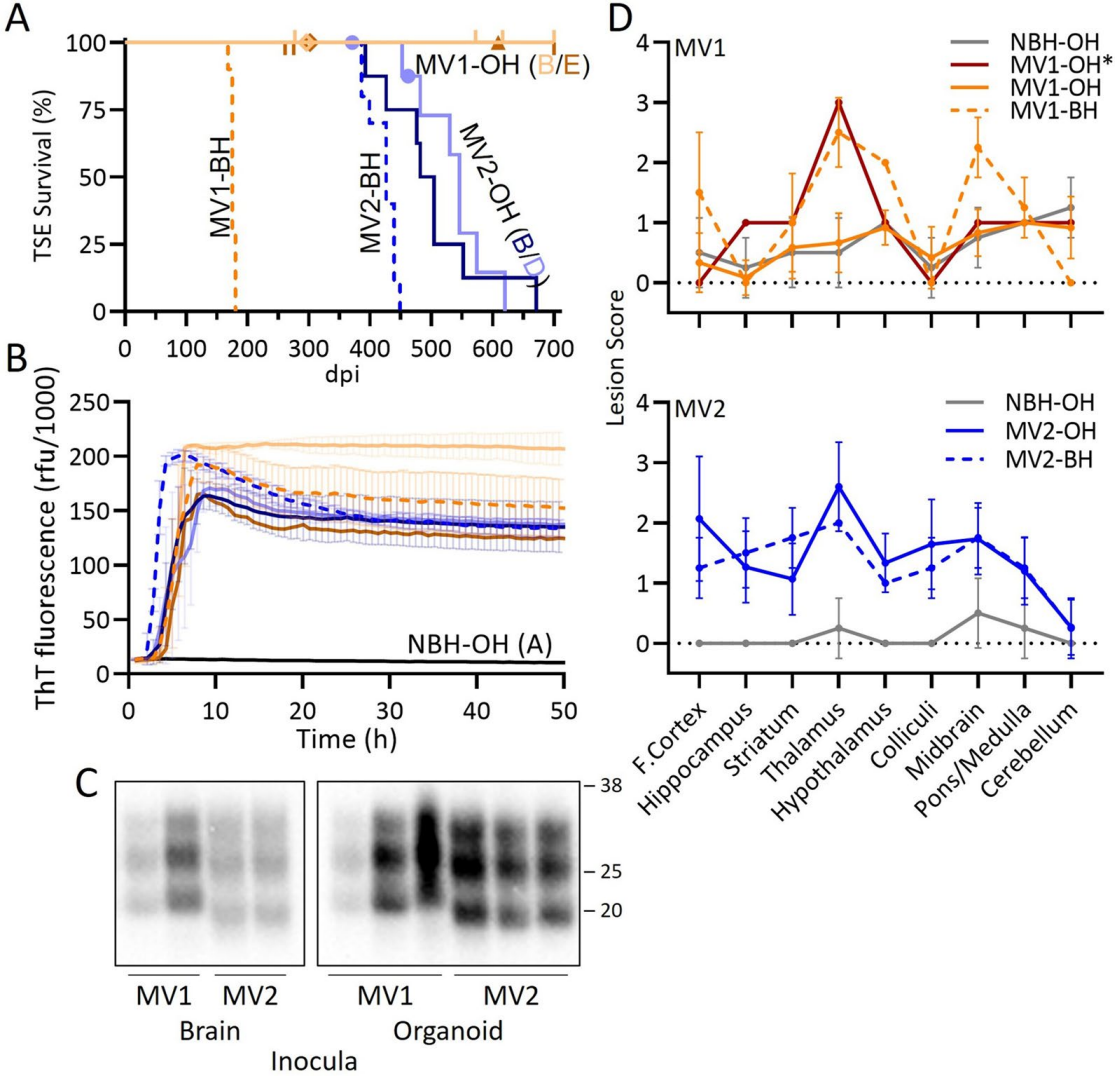
Organoid propagated sCJD prions are transmissible to mice and show similarity with the original brain homogenate prions. **A** Disease duration of mice inoculated with brain (dashed line) or organoid (solid line) homogenates. Tick marks represent non-TSE related deaths. ♦Two mice that were found dead without displaying signs of TSE but were positive by RT-QuIC and western blot. ▲Mouse euthanized for respiratory distress but was positive by IHC, RT-QuIC, and western blot. ●Two mice that displayed early signs of TSE but were euthanized for other reasons before full clinical manifestation. **B** Averaged RT-QuIC curves with S.D. from all RT-QuIC positive samples from each inoculation group. Colored curves correspond with the groups in panel a. **C** Western blots showing proteinase K (PK) resistant PrP (PrP^Res^)from BH (left panel) or OH (right panel) inoculated mice. To account for differences in PrP^Res^ signal due to the different deposition patterns between MV1 and MV2 inoculation groups, samples were loaded at different amounts to allow for better comparison of banding patterns. Numbers to the right indicate molecular weight in kDa. **D**. Lesion profiling from MV1 (orange), MV2 (blue) or NBH (gray; 481dpi top panel; ~ 700dpi bottom panel) BH (dashed line) or OH (solid line) inoculated mice. MV1-OH* shows the lesion profile of the one MV1-OH mouse with IHC staining that was also western blot positive for PrP^Res^. MV-OH groups show the average and S.D. of both respective organoid inoculum groups. Mann–Whitney U test results comparing lesion scores for each brain region and each inoculum can be found in Additional file 4: Table S1a. See Table 2 for number of replicates tested for each panel

**Fig. 2:**
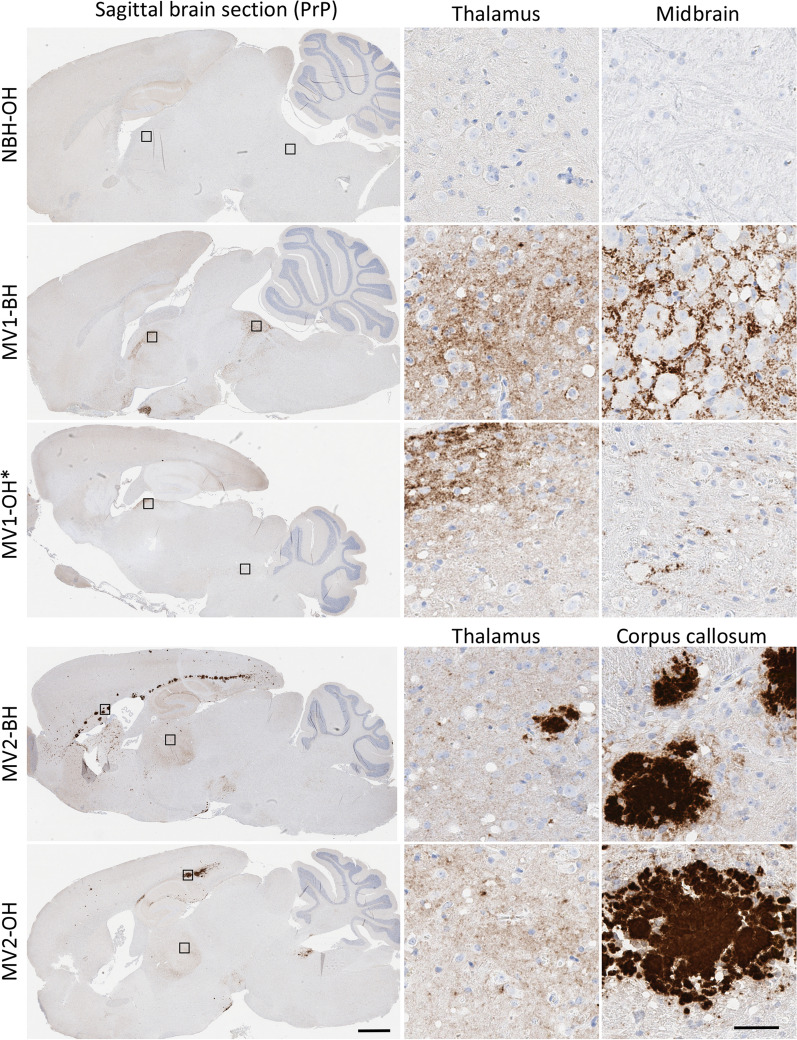
OH inoculated mice show PrP deposition consistent with the original BH subtype inoculated mice*.* Immunohistochemistry for prion protein (brown staining) showing distinct differences in deposition patterns between prion subtypes but similarities between mice inoculated with brain or organoid homogenate. Brain regions are indicated across the top and inocula are listed on the left of the image. Small boxes in the cross section correspond to the expanded images on the right. Scale bars indicate 1 mm and 50 µm for whole brain and insets, respectively

To investigate how true the prions propagated in the COs described in Groveman et al. [10] were to those from the original brain derived prions, homogenate from two of the organoids (MV genotype at codon 129) per subtype (reported in the original study as MV1-B, MV1-E, MV2-B, and MV2-D) and homogenates from the original MV-1 or -2 sCJD brains were inoculated into tg66 huPrP 129MM mice. The inocula and their prion titers (logSD_50_/ mg by RT-QuIC analysis) are shown in Table 1. Mice inoculated with sCJD brain homogenate succumbed to prion disease with a mean survival of 176 ± 4 days and 420 ± 23 days for the MV1-BH and MV2-BH inoculated mice, respectively (Fig. 1a, Table 2). Mice infected with the MV2 organoid homogenates (MV2-OH) all developed prion disease (mean survival of 501 ± 85 with MV2-OH (B) inoculum and 536 ± 56 with MV2-OH (D) inoculum) (Fig. 1a, Table 2) but showed a delay compared to the MV2-BH infected mice. While no MV1 organoid homogenate inoculated mice (MV1-OH) showed clear clinical signs of prion disease out to 700 dpi, two were found dead (296 and 302 dpi) and one was euthanized for other reasons (609 dpi) and their brain tissue found to be RT-QuIC positive, with PrP deposition detected by western blot and, in the one case where available, positive for immunohistochemical (IHC) hallmarks of prion disease (Fig. 1b–d, Fig. 2, Table 2). Although IHC staining was only positive in one MV1-OH infected mouse, cursory examination revealed lesion profiling very similar to the MV1-BH infected mice (Fig. 1d: MV1-OH*, Additional File 2: Figure S2). Similarly, the pattern of PrP deposition in the brain of this mouse followed that of the MV1-BH infections (Fig. 2) where deposition was primarily localized to the thalamus and midbrain. PrP^D^ staining was diffuse-synaptic in the thalamus and with prominent perineuronal granular staining in the midbrain. The remaining MV1-OH infected mice showed very few lesions, an observation similar to those in age-matched mice inoculated with NBH-OH (Fig. 1d, Additional File 2: Figure S2, Additional File 4: Table S1A). Analysis of the MV2-OH infection lesion profiles showed greater similarity with the MV2-BH than the MV1-BH inoculated mice (Fig. 1d, Additional File 2: Figure S2, Additional File 4: Table S1A). Additionally, the pattern of PrP deposition in the MV2-OH mice closely followed that of the MV2-BH infected mice (Fig. 2) where large PrP positive plaques were observed closely associated with the corpus callosum. In the samples positive by western blot, banding patterns similar to the original inocula [10] were observed for both BH and OH infected animals, with the MV1 samples migrating more slowly through the gels (Fig. 1c).

**Table 1:** Initial infection inocula

Inocula for P1	SD50/mg	Blot
MV1-BH	1.8 × 10^6^	+
MV1-OH (B)	1.6 × 10^3^	−
MV1-OH (E)	≤ 2.8 × 10^3^	−
MV2-BH	1.0 × 10^6^	+
MV2-OH (B)	5.0 × 10^5^	+
MV2-OH (D)	1.6 × 10^5^	+
NBH-OH (A)	NA	−

P1: passage 1; SD50/mg: seeding dose 50% as determined by RT-QuIC (see methods); Blot: results of western blotting for PrP^Res^

**Table 2:** Summary of first passage findings

Inocula	Clinical	DPI	IHC	RT-QuIC	Blot
MV1-BH	10/10	176 ± 4	4/4	3/3	3/3
MV1-OH (B)	0/9	NA	0/7	1/9	1/1
MV1-OH (E)	0/5	NA	1/6	2/7	2/2
MV2-BH	10/10	420 ± 23	4/4	3/3	3/3
MV2-OH (B)	8/8	501 ± 85	8/8	8/8	1/1
MV2-OH (D)	7/9	536 ± 56	8/8	9/9	3/3
NBH-OH (A)	0/8	NA	0/8	0/8	0/3

Clinical: mice showing terminal prion disease. Mice found dead or euthanized for non-TSE related reasons were not counted towards the total, although tissue may have been used for other assays; *DPI* days post inoculation to euthanasia, *IHC* immunohistochemistry for PrP deposition, *RT-QuIC* results of real-time quaking induced conversion assay; Blot: results of western blotting for PrP^Res^; x/y indicate total positive out of total tested. Letters in parenthesis are identifiers to link the inoculating organoids to the original study [10]

### Sub-passaged prions from organoid homogenate inoculated mice cause disease with characteristics of the original human brain subtype inocula

**Fig. 3:**
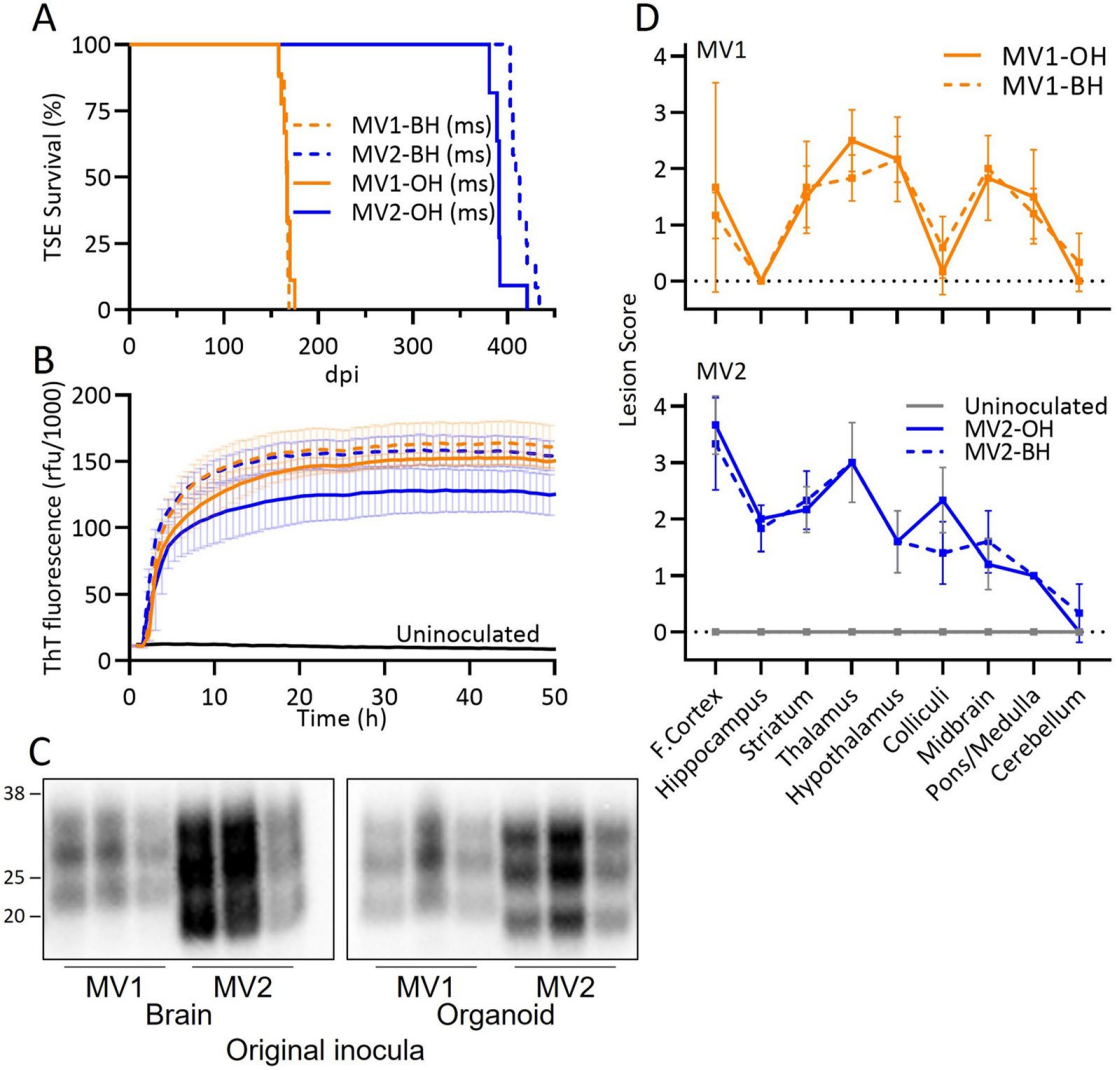
Sub-passage from OH inoculated mice cause disease with traits consistent with that from the original BH subtype inocula*.* **A** Disease duration of second pass mice inoculated with MV-BH (ms) (dashed lines) or MV-OH (ms) (solid lines) homogenates. **B** RT-QuIC curves displayed as an average with S.D. of all mice tested for each group (see Table 4 for number of replicate brains tested). Colored curves correspond with the groups in panel a. **C** Western blots showing proteinase K resistant PrP in the brains of sub-passaged mice inoculated with MV-BH (ms) (left panel) or MV-OH (ms) (right panel) homogenate. To account for differences in PrP^Res^ signal due to the different deposition patterns between MV1 and MV2 inoculation groups, samples were loaded at different amounts to allow for better comparison of banding patterns. Numbers to the left indicate molecular weight in kDa. **D**. Lesion profile comparisons of MV-BH (ms) (solid line) and MV-OH (ms) (dashed line) inoculated mice are shown for MV1 (top panel) and MV2 (bottom panel) groups as well as young uninoculated mice (177 dpi; bottom panel, gray) controls. Shown are the mean and S.D. Mann–Whitney U test results comparing lesion scores for each brain region and each inoculum can be found in Additional File 4: Table S1B. See Table 4 for number of replicates tested for each panel

**Fig. 4:**
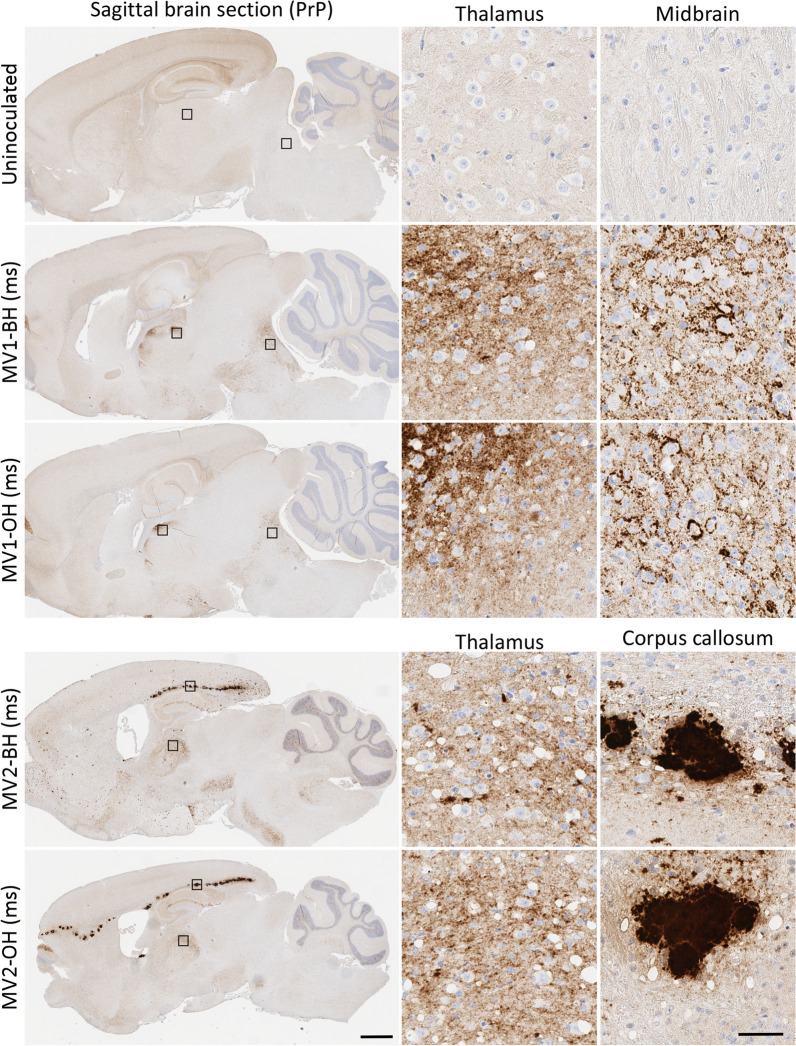
Second pass mice show PrP deposition consistent with original inoculum subtype. Immunohistochemistry of second pass mice shows maintained differences in PrP deposition patterns between prion subtypes and similarities between MV-BH (ms) and MV-OH (ms) inoculated mice. Small boxes in the cross section correspond to the expanded images on the right. Brain regions are indicated across the top and inocula are listed on the left of the image. Scale bars indicate 1 mm and 50 µm for whole brain and insets, respectively

Since the lower prion seeding titers of the organoid inocula (Table 1) likely account for the differences in attack rates, incubation periods, RT-QuIC, western blot and/or IHC positivity, we performed a second passage using western blot and IHC positive brains from the OH and BH infected mice. The sub-passaged inocula and their prion titers (logSD_50_/mg by RT-QuIC analysis) are shown in Table 3. In this subpassage all infected mice succumbed to prion disease and the survival times clearly grouped as type 1 or type 2 regardless of whether the original inoculum had come from brain homogenate (MV-BH (ms)) or organoids (MV-OH (ms)) (“ms” indicates passage through mice; Fig. 3a). In the first passage, the MV2-OH mice had a longer survival time than the MV2-BH mice (Fig. 2a); however, in the second passage the MV2-OH (ms) mice had a slightly shorter survival time than the MV2-BH (ms) mice (Fig. 3a). This subtle difference can likely be attributed to the differences in prion titer of the inoculum used at each passage (Table 1 and 3). All tested brains were positive by RT-QuIC (Fig. 3b, Table 4) and were positive for PrP^Res^ by western blotting (Fig. 3c, Table 4). The banding profile of the western blots also indicated that the mobility associated with the different subtypes had been preserved. IHC analyses showed clear differences between the MV1 and MV2 infections with only diffuse-synaptic and perineuronal granular deposition of PrP in the MV1 infections compared with large PrP^D^ plaques in the corpus callosum, smaller widespread plaques, coarse granular staining and a lesser amount of diffuse PrP^D^ in the MV2 infections (Fig. 4). Further examination of the H&E stained brains revealed that, while not perfectly overlapping, MV-OH (ms) and MV-BH (ms) inocula produced very similar lesion profiles (Fig. 3d and Additional File 3: Figure S3, Additional File 1: Table S1B).

**Table 3:** Passage 2 inocula

Inocula for P2	DPI	IHC	Blot	SD50/mg
MV1-BH (ms)	180	+	+	8.9 × 10^6^
MV1-OH (ms)	609^#^	+	+	5.0 × 10^6^
MV2-BH (ms)	421	+	+	3.8 × 10^7^
MV2-OH (ms)	671	+	+	2.8 × 10^7^

P2: passage 2; Characteristics of mice used for second passage inocula including: DPI: days post inoculation to euthanasia; IHC: immunohistochemistry for PrP deposition; Blot: results of western blotting for PrP^Res^; SD50/mg: seeding dose 50% as determined by RT-QuIC (see methods); ^#^Mouse was euthanized for non-TSE related reasons before developing clear clinical signs

**Table 4:** Summary of passage 2 results

Inocula	Clinical	DPI	IHC	RT-QuIC	Blot
MV1-BH (ms)	8/8	166 ± 3	6/6	3/3	4/4
MV1-OH (ms)	8/8	166 ± 5	6/6	3/3	6/6
MV2-BH (ms)	12/12	413 ± 11	6/6	3/3	6/6
MV2-OH (ms)	12/12	392 ± 10	6/6	3/3	6/6
Uninfected	0/4	177 ± 0	0/4	0/4	0/5

Clinical: mice showing terminal prion disease. *DPI* days post inoculation to euthanasia, *IHC* immunohistochemistry for PrP deposition; RT-QuIC: results of Real-Time Quaking Induced Conversion assay; Blot: results of western blotting for PrP^Res^; x/y indicate total positive out of total tested

## Discussion

Human cerebral organoids offer a powerful model of brain tissue in a dish that can be used to investigate neurodegenerative disorders. Our previous work showed that cerebral organoids could be infected with sCJD prions derived from human brain tissue. However, as organoids represent, but are not actual brain tissue, it was imperative to determine if the prions propagated within them were the original subtype or adaptations. This knowledge is necessary to properly understand the data produced and its potential limitations.

In our initial study we noted a change in the glycosylation pattern of the PrP^Res^ deposited in the organoids which we postulated could be a sign of strain adaptation. However, recent studies on RML [30] and anchorless RML [31] prions, which lack a glycophosphatidylinositol anchor and are deficient in N-linked glycans, show nearly identical atomic structures. This suggests that while glycosylation differences may depend on the host environment, the subtype information is encoded in the protein backbone conformation and is not materially affected by the addition of glycans. Indeed, our current data show that despite the shift in glycosylation state the prions generated by infection of the organoids are both infectious when inoculated into mice and maintain their original subtype characteristics. Incubation periods, lesion profiles, patterns of PrP^D^ deposition throughout the brain, and PrP^Res^ electrophoretic mobility profiles all suggest that the organoid propagated prions do not deviate substantially from the brain-derived prions with which they were inoculated. In fact, despite having higher titers of prion seeding activity, the MV2 derived homogenates had longer incubation periods in mice than the MV1 derived homogenates, consistent with the clinical presentation of these subtypes in humans [32].

The incubation periods of different type 1 and 2 brain homogenates in humanized mice vary in the literature [23, 33–35]. This is at least partially due to the mouse strain, which may have different PrP^C^ expression levels, and classification system used to define the subtypes. However, the strain characteristics of these prion subtypes can be propagated with little change upon subpassage, regardless of the 129 polymorphism of the inoculum or mouse [36]. In our work, the brains originally used for inoculation [10] gave widely divergent, subtype dependent, incubation periods and plaque pathologies in the infected mice, making the subtype differences more apparent. This allowed us to more easily determine that each of the original infecting prions had not been substantially changed by passage through the organoid.

Although the MV1-OH inoculated mice showed an extended incubation period on first passage which was substantially shortened on subpassage to match that of the MV1-BH inoculated mice, we do not attribute this to a strain adaptation. Instead, it is more likely that the prion titer is responsible for the difference in incubation periods [37]. The original MV1-OH inoculum had > 1000-fold less prion seeding activity than the MV1-BH inocula (Table 1). At the endpoints of the first passage (609 for the mouse that was subsequently used for subpassage vs 180 dpi, respectively), the MV1-OH inoculated mouse brain tissue had a prion seeding titer nearly equivalent to the MV1-BH inoculated mouse brain tissue used for subpassage (Table 3). Aside from the incubation period difference, the other disease characteristics in the mouse with subclinical disease closely matched the MV1-BH inoculated mice. Additionally, since the incubation periods in the MV2 inoculated animals closely followed their respective titers as well (Tables 1 and 3), we conclude that the change in incubation period in the MV1-OH mice from first to second passage is likely a result of prion titer and not indicative of a strain adaptation.

There also remains the possibility that, as COs are non-dividing cultures, remnant human brain tissue derived PrP^D^ may still be present from the initial inoculation and be responsible for the infection in mice. Indeed, residual inocula is a critical concern when studying infection in COs. In our initial study ([10] and Additional File 1: Figure S1), we followed the residual inoculum, as detected by RT-QuIC, and observed the complete disappearance around 25–30 dpi followed by the re-emergence at later time points of RT-QuIC positivity to levels greater than those immediately following inoculation. Since the RT-QuIC assay has been demonstrated to be more sensitive than animal bioassay and able to detect sub-infectious levels of PrP^D^ [28, 38–40], the complete loss of RT-QuIC detection of the inocula suggests that, if present, the residual inocula in the organoids would not be sufficient to cause disease in animals, even following subpassage [41]. Instead, this suggests that organoid derived PrP^D^ is responsible for both MV1 and MV2 infections despite the long incubation period and low detection in the first passage MV1-OH inoculated mice. While we don’t yet know if serial subpassages through COs would eventually result in deviation from the original inoculating subtype, or whether inoculation into mice may have helped stabilize the strains [42, 43], we now know that the characteristics of the PrP^D^ generated in the organoid and the resulting infection cause by initial inoculation with a particular subtype are maintained.

The maintenance of the subtype characteristics in the infected organoids is beneficial for investigating prion pathogenesis in different neuronal populations. As previously stated, investigation into how the different subtypes cause different prion disease phenotypes and selectively attack different neuronal subsets has been limited by the lack of a human-derived model in which propagation of different subtypes could be faithfully reproduced. With confidence in the reproduction of the prion subtype features within the organoids, we may be able to address the questions as to why the disease proceeds faster in certain subtypes and begin to mechanistically analyze subtype-dependent pathogenesis. Furthermore, we may be able to extend this investigation to other prion subtypes as well as prions associated with hereditary disease. While ourselves and others have reported no evidence of propagating prions in human iPSC derived organoids and neurons from several hereditary disease-causing mutations [25, 44, 45], other pathological changes have been observed such as tau accumulation [45] and neuronal network dysfunction [46]. Infection of organoids with hereditary PrP mutations may reveal the different pathways by which these prions produce their own manifestation of prion disease.

A further advantage of the organoid system that benefits from the maintenance of subtype properties is the potential use for screening therapeutics, as we have previously demonstrated [26]. An ideal drug would be effective against all prion disease subtypes, but this is not always the case. Now, with a model that can maintain the sCJD subtype, it may be possible to discern if a potential therapeutic strategy will work across all subtypes or be more effective in one type over another. It should be noted that, like all disease models, organoids are not without their limitations. We have discussed many of these, the impact they have on interpretation of results, and possible improvements to the system previously [13, 15]. However, the closer the system can come to representing genuine human diseases the better the chance we can translate our findings into meaningful interventions.

## Conclusions

Protein misfolding diseases are characterized by the aggregation of self-propagating misfolded proteins that spread throughout the nervous system. Structural perturbations of these misfolded proteins give rise to unique strains, each with their own pathological characteristics. Deeper understanding of this strain phenomena can help to gain better insight into the mechanisms of neurodegeneration. Herein we provide evidence that cerebral organoids can be used to faithfully propagate specific strains of prion disease as confirmed by transmission into the gold standard animal bioassay. The original infecting prion appears to be the predominant determinant of the prion propagated within the human cerebral organoids with little if any modification by the organoid environment. As the first in vitro fully human model of neurodegenerative disease that is capable of propagating and transmitting disease while maintaining subtype characteristics, cerebral organoids provide a critical platform for better understating prion-induced neurodegeneration.

## Additional files

**Additional file 1. Figure S1**. Summary of previous results and preparation of organoid inocula. A. Schematic of organoid maturation, inoculation, collection, and inoculation into mice. Cerebral organoids were generated from donor human iPSCs that were heterozygous at codon 129 (MV) as described in Lancaster and Knoblich [11] and maintained in agitated culture under standard incubator conditions (37˚C, 5% CO_2_, humidified). Organoids were allowed to mature for 140 days to ensure populations of mature oligodendrocytes and astrocytes were present. Following infection with MV1 and MV2 sCJD brain homogenate inocula, organoids were collected at the indicated time points. Whole organoids from the final time point were homogenized by motorized pestle to 10% w/v in PBS. Homogenates were processed and inoculated into tg66 mice that overexpress human PrP^C^ homozygous for methionine at codon 129 as described in methods. Do = days old, dpi = days post infection. B. Summary of organoid RT-QuIC results showing the loss of detectable seeding activity from the inoculum by 28 dpi and the subsequent re-emergence of de novo, organoid derived, PrP^D^ seeding activity. C. Mass of the organoids from the final collection prior to homogenization to 10% w/v. Organoids used for inocula are indicated by their corresponding letter code. D. Schematic of glycosylation patterns observed in brain and organoid derived PrP^C^ (top) and PrP^Res^ (bottom). Figures modified from [10].

**Additional file 2. Figure S2**. Regions and severity of spongiform change consistent between OH and BH inoculated mice for each subtype*.* Representative H&E staining of different brain regions from mice inoculated with MV-BH or MV-OH homogenates showing the patterns of spongiform change. Brain regions are listed across the top and inocula are listed on the left of the image. Arrows indicate regions of large PrP plaques. Scale bar indicates 50 µm.

**Additional file 3. Figure S3**. Sub-passaged mice show regions and severity of spongiform change consistent between mice originally inoculated with OH or BH for each subtype*.* Representative H&E staining of different brain regions from sub-passaged mice inoculated with MV-BH (ms) and MV-OH (ms) homogenates showing the patterns of spongiform change. Arrows indicate regions of large PrP plaques. Inocula are listed on the left of the image. Scale bar indicates 50 µm.

**Additional file 4. Table S1**. Brain regions showing statistically significant (p<0.05) differences in lesion scores by Mann-Whitney U test.

## Data Availability

The datasets used and/or analyzed during the current study are available from the corresponding author on reasonable request.

## Abbreviations

PrD: Prion disase
PrP: Prion protein
PrP^Res^: Protease resistant PrP
sCJD: Sporadic Creutzfeldt–Jacob disease
PrP^D^: Disease-associated PrP
PrP^C^: Cellular PrP
M: Methionine
V: Valine
BH: Brain homogenate
OH: Organoid homogenate
MV-BH: CJD (either type 1 or 2) infected BH
MV-OH: CJD (either type 1 or 2) infected OH
IHC: Immunohistochemistry
MV-BH (ms): Mouse BH originally infected with CJD infected BH
MV-OH (ms): Mouse BH originally infected with CJD infected OH
