# Supplementary figures and images for: Sporadic Creutzfeldt–Jakob disease infected human cerebral organoids retain the original human brain subtype features following transmission to humanized transgenic mice
Source: Acta Neuropathol Commun. 2023 Feb 14;11:28. doi: 10.1186/s40478-023-01512-1 (PMC9930245; doi:10.1186/s40478-023-01512-1)

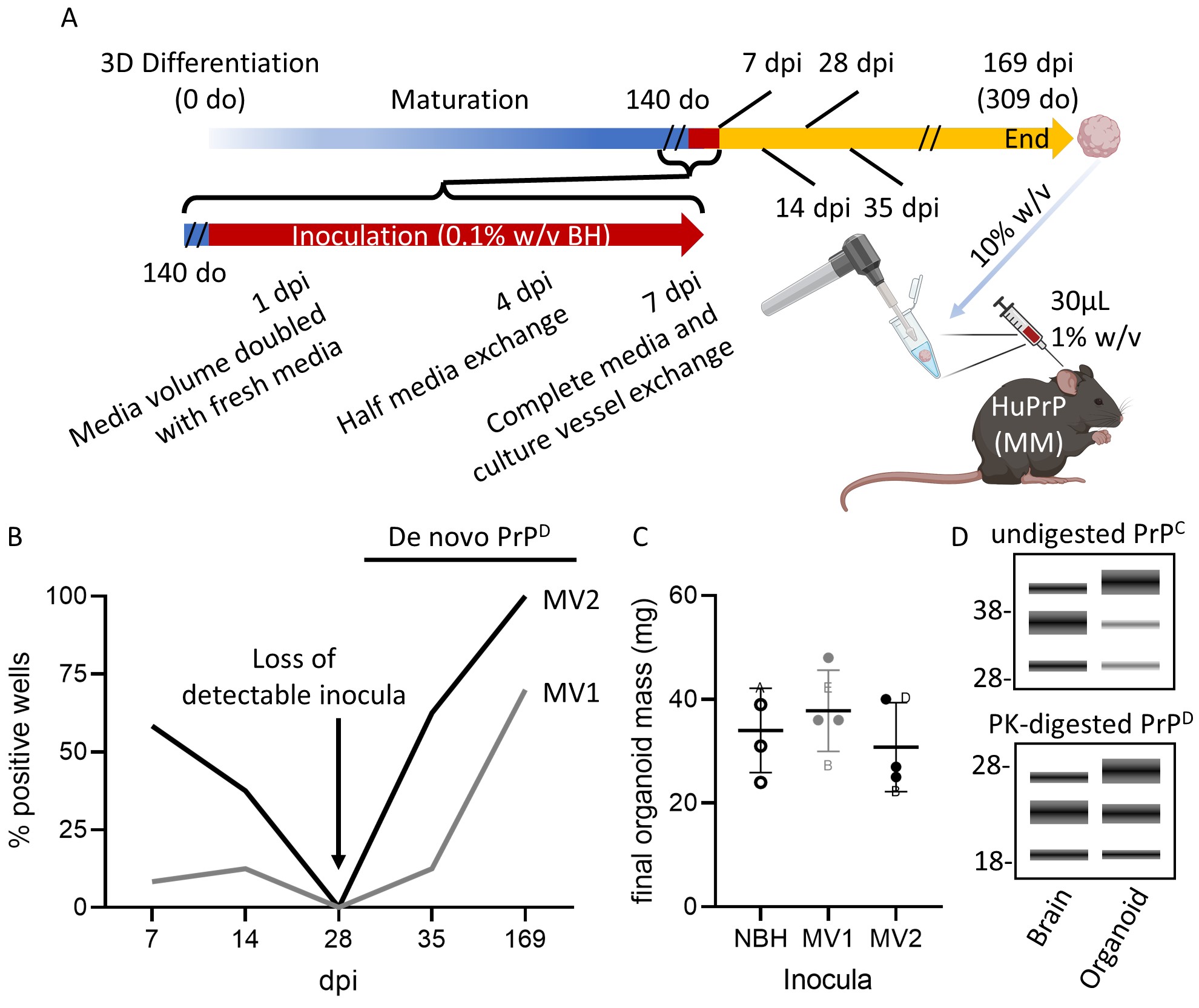

Supplement: Supplementary file 1 — Additional file 1. Figure S1. Summary of previous results and preparation of organoid inocula. A. Schematic of organoid maturation, inoculation, collection, and inoculation into mice. Cerebral organoids were generated from donor human iPSCs that were heterozygous at codon 129 (MV) as described in Lancaster and Knoblich [11] and maintained in agitated culture under standard incubator conditions (37˚C, 5% CO2, humidified). Organoids were allowed to mature for 140 days to ensure populations of mature oligodendrocytes and astrocytes were present. Following infection with MV1 and MV2 sCJD brain homogenate inocula, organoids were collected at the indicated time points. Whole organoids from the final time point were homogenized by motorized pestle to 10% w/v in PBS. Homogenates were processed and inoculated into tg66 mice that overexpress human PrPC homozygous for methionine at codon 129 as described in methods. Do = days old, dpi = days post infection. B. Summary of organoid RT-QuIC results showing the loss of detectable seeding activity from the inoculum by 28 dpi and the subsequent re-emergence of de novo, organoid derived, PrPD seeding activity. C. Mass of the organoids from the final collection prior to homogenization to 10% w/v. Organoids used for inocula are indicated by their corresponding letter code. D. Schematic of glycosylation patterns observed in brain and organoid derived PrPC (top) and PrPRes (bottom). Figures modified from [10]. [file 40478_2023_1512_MOESM1_ESM.jpg]

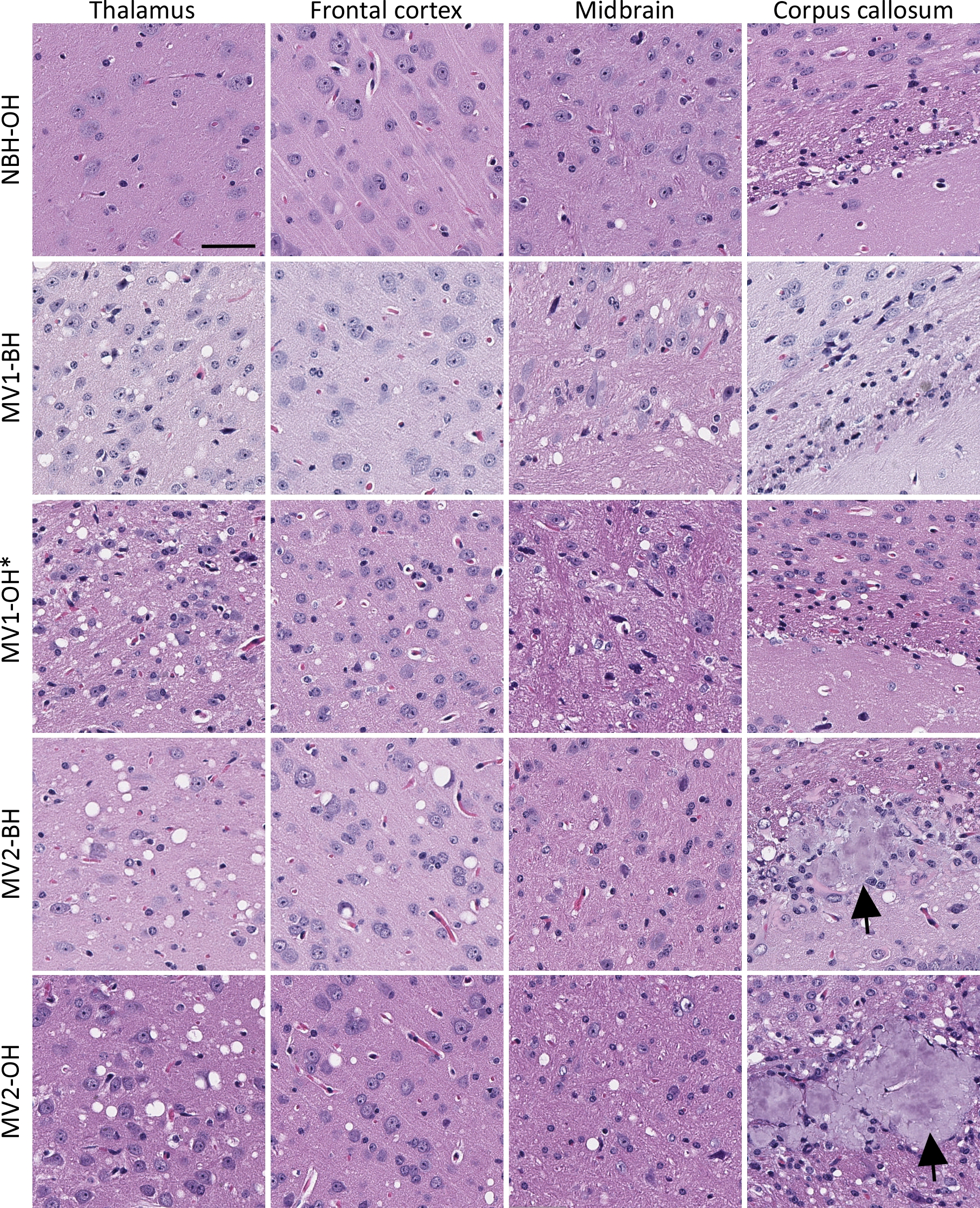

Supplement: Supplementary file 2 — Additional file 2. Figure S2. Regions and severity of spongiform change consistent between OH and BH inoculated mice for each subtype. Representative H&E staining of different brain regions from mice inoculated with MV-BH or MV-OH homogenates showing the patterns of spongiform change. Brain regions are listed across the top and inocula are listed on the left of the image. Arrows indicate regions of large PrP plaques. Scale bar indicates 50 µm. [file 40478_2023_1512_MOESM2_ESM.jpg]

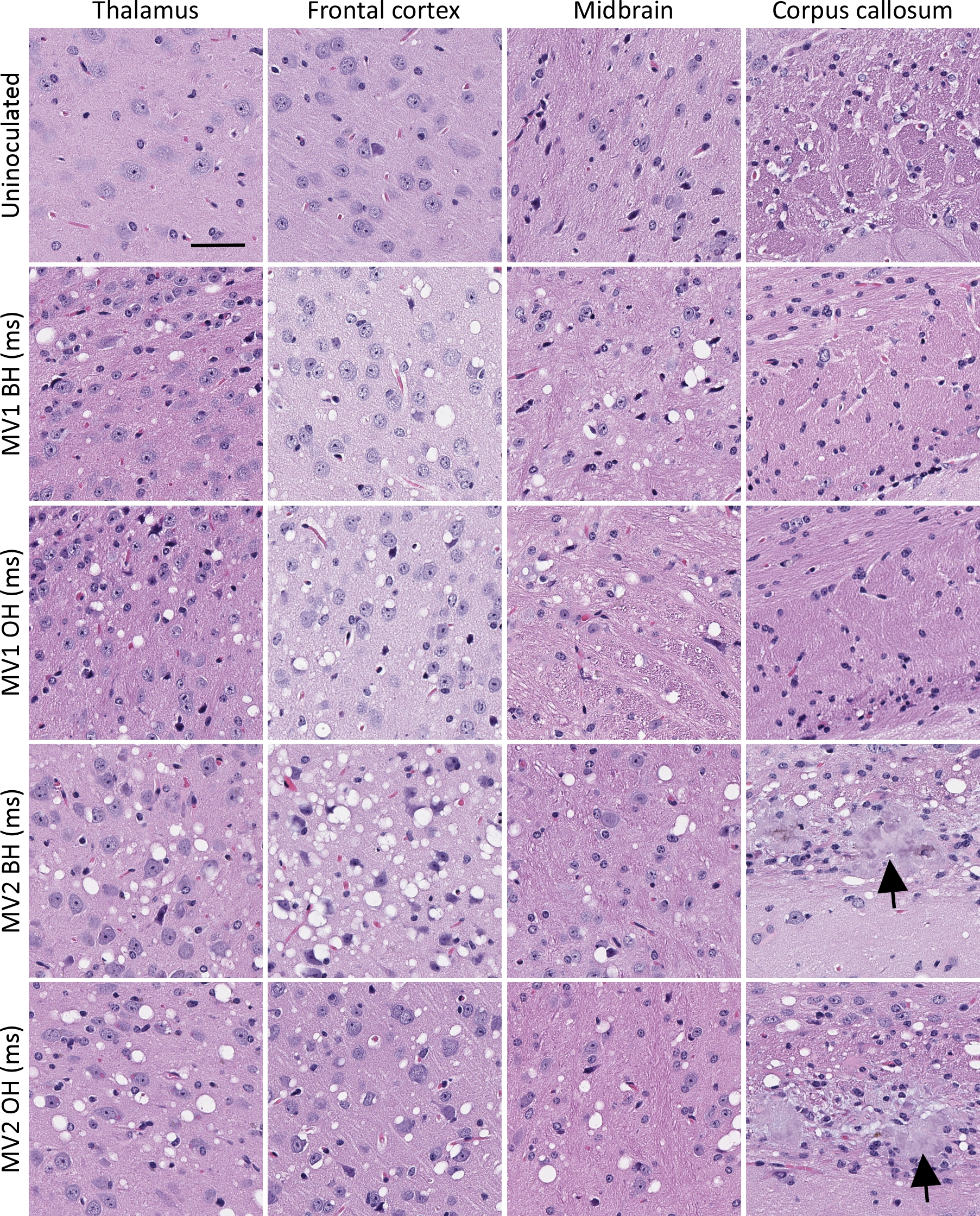

Supplement: Supplementary file 3 — Additional file 3. Figure S3. Sub-passaged mice show regions and severity of spongiform change consistent between mice originally inoculated with OH or BH for each subtype. Representative H&E staining of different brain regions from sub-passaged mice inoculated with MV-BH (ms) and MV-OH (ms) homogenates showing the patterns of spongiform change. Arrows indicate regions of large PrP plaques. Inocula are listed on the left of the image. Scale bar indicates 50 µm. [file 40478_2023_1512_MOESM3_ESM.jpg]
