# Supplementary material for: Sporadic Creutzfeldt–Jakob disease infected human cerebral organoids retain the original human brain subtype features following transmission to humanized transgenic mice
Source: Acta Neuropathol Commun. 2023 Feb 14;11:28. doi: 10.1186/s40478-023-01512-1 (PMC9930245; doi:10.1186/s40478-023-01512-1)
Supplement: Supplementary file 4 — Additional file 4. Table S1. Brain regions showing statistically significant (p<0.05) differences in lesion scores by Mann-Whitney U test. [file 40478_2023_1512_MOESM4_ESM.docx]

**Supplementary Table 1:** Brain regions showing statistically significant (p<0.05) differences in lesion scores by Mann-Whitney U test

**A.** First passage mice

| MV1-BH | ^#^FC, Thal, Hyp, Mb, P/M |  |  |  |
| --- | --- | --- | --- | --- |
| MV1-OH | NS | FC, Thal, Hyp, Mb, Cb |  |  |
| MV2-BH | FC, Hip, Str, Thal, Hyp, Col | Hip, Hyp, Col | FC, Hip, Str, Thal, Mb |  |
| MV2-OH | FC, Hip, Str, Thal, Hyp, Col, Mb, P/M | Hip, Hyp, Col | FC, Hip, Thal, Hyp, Col, Mb, Cb | NS |
|  | *NBH | MV1-BH | MV1-OH | MV2-BH |

**B.** Second passage mice

| MV1-BH | FC, Str, Thal, Hyp, Mb, P/M |  |  |  |
| --- | --- | --- | --- | --- |
| MV1-OH | Str, Thal, Hyp, Mb, P/M | NS |  |  |
| MV2-BH | FC, Hip, Str, Thal, Hyp, Col, P/M | FC, Hip, Thal, Col | Hip, Col |  |
| MV2-OH | FC, Hip, Str, Thal, Hyp, Col, P/M | FC, Hip, Str, Thal, Hyp, Col | FC, Hip, Str, Col | NS |
|  | NBH | MV1-BH | MV1-OH | MV2-BH |

^#^FC: Frontal Cortex, Hip: Hippocampus, Str: Striatum, Thal: Thalamus, Hyp: Hypothalamus, Col: Colliculi, Mb: Midbrain, P/M: Pons/Medulla, Cer: Cerebellum, NS: not significant

*Each experimental group was compared with the set of control mice that was most closely age matched: P1 and P2 MV1-BH and P2 MV1-OH were compared with 177 dpi uninoculated control mice; P1 MV1-OH was compared with 700 dpi NBH-OH inoculated control mice; P1 and P2 MV2-BH and MV2-OH were compared with 481 dpi NBH-OH inoculated control mice
